# *β*-Arrestins promote podocyte injury by inhibition of autophagy in diabetic nephropathy

**DOI:** 10.1038/cddis.2016.89

**Published:** 2016-04-07

**Authors:** J Liu, Q X Li, X J Wang, C Zhang, Y Q Duan, Z Y Wang, Y Zhang, X Yu, N J Li, J P Sun, F Yi

**Affiliations:** 1 Department of Pharmacology, Shandong University School of Medicine, Jinan 250012, China; 2 Department of Nephrology, Union Hospital, Tongji Medical College, Huazhong University of Science and Technology, Wuhan 430022, China; 3 Department of Physiology, Shandong University School of Medicine, Jinan 250012, China; 4 Department of Pharmacology and Toxicology, Medical College of Virginia, Virginia Commonwealth University, Richmond, VA 23298, USA; 5 Department of Biochemistry and Molecular Biology, Shandong University School of Medicine, Jinan 250012, China; 6 Institute of Nephrology, Shandong University, Jinan 250012, China

## Abstract

*β*-Arrestins are multifunctional proteins originally identified as negative adaptors of G protein-coupled receptors (GPCRs). Emerging evidence has also indicated that *β*-arrestins can activate signaling pathways independent of GPCR activation. This study was to elucidate the role of *β*-arrestins in diabetic nephropathy (DN) and hypothesized that *β*-arrestins contribute to diabetic renal injury by mediating podocyte autophagic process. We first found that both *β*-arrestin-1 and *β*-arrestin-2 were upregulated in the kidney from streptozotocin-induced diabetic mice, diabetic *db/db* mice and kidney biopsies from diabetic patients. We further revealed that either *β*-arrestin-1 or *β*-arrestin-2 deficiency (*Arrb1*^*−/−*^ or *Arrb2*^*−/−*^) ameliorated renal injury in diabetic mice. *In vitro*, we observed that podocytes increased both *β*-arrestin-1 and *β*-arrestin-2 expression levels under hyperglycemia condition and further demonstrated that *β*-arrestin-1 and *β*-arrestin-2 shared common mechanisms to suppress podocyte autophagy by negative regulation of ATG12–ATG5 conjugation. Collectively, this study for the first time demonstrates that *β*-arrestin-1 and *β*-arrestin-2 mediate podocyte autophagic activity, indicating that *β*-arrestins are critical components of signal transduction pathways that link renal injury to reduce autophagy in DN. Modulation of these pathways may be an innovative therapeutic strategy for treating patients with DN.

Although multiple approach targeting blood pressure, inflammation and the levels of glucose and insulin has been used in clinical trials, there is no effective therapy available to fully prevent the onset and progression of diabetic nephropathy (DN). Therefore, it is urgent to identify new therapeutic targets and develop an effective strategy for the treatment of DN. Autophagy is a catabolic process that degrades damaged proteins and organelles in mammalian cells and has a vital role in maintaining cellular homeostasis. To date, 36 autophagy-related genes (ATGs) are identified, which have revealed dynamic and diverse mechanisms to orchestrate autophagy induction, autophagosomal membrane nucleation, elongation, closure and maturation.^1, 2^ Emerging evidence has indicated that autophagy regulates many critical aspects of normal and disease conditions in the kidney^3^ and is especially important for the maintenance of postmitotic cells, such as podocytes.^4^ Podocyte-specific deletion of ATG5 leads to proteinuria and glomerulopathy in aging mice.^3, 5^ Mice with podocyte-specific deletion of class III phosphoinositide 3-kinase (PI3K) vacuolar protein sorting 34 (VPS34), which is essential to initiate autophagy, exhibits substantial vacuolization, foot process effacement and progressive glomerulosclerosis.^6^ These findings indicate that autophagy is a key homeostatic mechanism to maintain podocyte integrity and function. Although studies have implicated that autophagy machinery is involved in the pathogenesis of DN,^7, 8^ the precise roles of autophagy and related regulatory mechanisms are largely unknown.

*β*-Arrestins (Arrbs) are multifunctional proteins originally identified as negative adaptors of G protein-coupled receptors (GPCRs) by regulation of their desensitization and internalization. Recent studies have also demonstrated that *β*-arrestins function to activate signaling cascades independently of GPCR activation.^9^ They can act as scaffold proteins that aggregate various intracellular proteins to initiate complex signaling pathways, such as mitogen-activated protein kinase, c-Jun N-terminal kinase and nuclear factor-kappa B cascades. The family of *β*-arrestins consists of four members, arrestins 1 and 4 that are exclusively confined to the cones and rodes of the retina and arrestins 2 and 3 (*β*-arrestin-1 (Arrb1) and *β*-arrestin-2 (Arrb2), respectively, that are universally expressed in all mammalian tissues.^10^ In the kidney, *β*-arrestin-2 can mediate nephrin endocytosis and impair slit diaphragm integrity.^11, 12^ In this study, we identify for the first time that *β*-arrestins mediate podocyte autophagy in DN that provides a novel molecular mechanism of slit diaphragm distortion. A better understanding of the function of *β*-arrestins in the kidney will provide unexpected opportunities for developing new therapies for DN.

## Results

### Upregulation of *β*-arrestin-1 and *β*-arrestin-2 in the kidney from STZ-induced diabetic mice, diabetic *db/db* mice and kidney biopsies from diabetic patients

**Figure 1:**
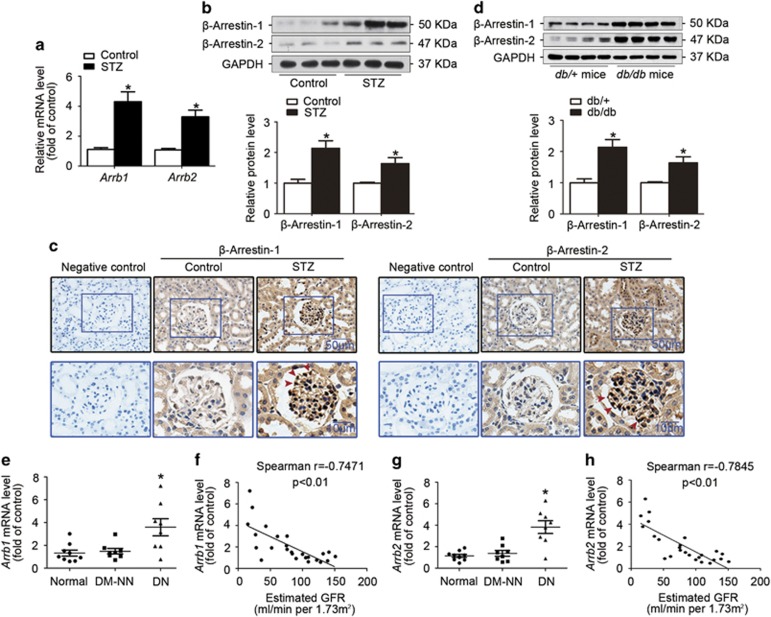
Upregulation of *β*-arrestin-1 and *β*-arrestin-2 in the kidney from STZ-induced diabetic mice, diabetic *db/db* mice and kidney biopsies from diabetic patients. (**a**) Relative mRNA levels of *Arrb1* and *Arrb2* in the kidney from STZ-induced diabetic mice (mean±S.E.M.). (**b**) Representative western blotting gel documents and summarized data showing the relative protein levels of *β*-arrestin-1 and *β*-arrestin-2 in the kidney from STZ-induced diabetic mice (mean±S.E.M.). (**c**) Representative images are from ISH of *β*-arrestin-1 and *β*-arrestin-2 in the kidney from STZ-induced diabetic mice (arrowheads: representative podocytes). Scramble probes were used as negative controls. (**d**) Representative western blotting gel documents and summarized data showing the relative protein levels of *β*-arrestin-1/2 in the kidney from diabetic *db/db* mice and their genetic control *db/+* mice (mean±S.E.M.). (**e**) Relative mRNA levels of *Arrb1* in from normal subjects (*n*=9) and patients with DN (*n*=8) or DM-NN (*n*=8) (mean±S.E.M.). (**f**) Negative correlation (Spearman *r*=−0.7471, *P*<0.05) between *Arrb1* mRNA levels and estimated glomerular filtration rate (eGFR) in all subjects. (**g**) Relative mRNA levels of *Arrb2* in the renal biopsies from different patients (mean±S.E.M.). (**h**) Negative correlation (Spearman *r*=−0.7845, *P*<0.05) between *Arrb2* mRNA levels and eGFR in all subjects. *P<0.05 *versus* normal subjects

As shown in Supplementary Table S1, STZ-induced diabetic mice had hyperglycemia and lower body weight compared with their non-diabetic counterparts, and no difference in blood pressure was observed among these groups. Real-time RT-PCR (Figure 1a) and western blotting (Figure 1b) analyses showed that both *β*-arrestin-1 and *β*-arrestin-2 were upregulated in the kidney from diabetic mice, which was further confirmed in paraffin-embedded sections of kidney tissues by *in situ* hybridization (ISH; Figure 1c) or immunohistochemical staining (Supplementary Figure S1a). We also observed the upregulation of *β*-arrestin-1 and *β*-arrestin-2 in the kidney in another *in vivo* model of diabetes, the *db/db* mice, indicating that *β*-arrestin-1 and *β*-arrestin-2 may be the common pathogenic factors in DN (Figure 1d). In consistent with the changes in animal studies, real-time RT-PCR (Figures 1e–g) and immunohistochemical staining (Supplementary Figure S1b) further confirmed the upregulation of *β*-arrestin-1 and *β*-arrestin-2 in the human diabetic renal tissues compared with normal controls and diabetic patients without nephropathy (DM-NN). We further found that both *Arrb1* (Spearman *r*=−0.7471, *P*<0.01; Figure 1f) and *Arrb2* (Spearman *r*=−0.7845, *P*<0.01; Figure 1h) mRNA levels were negatively correlated with estimated glomerular filtration rate in all the available subjects individually.

### Either *Arrb1* or *Arrb2* deficiency ameliorated renal injury in diabetic mice

**Figure 2:**
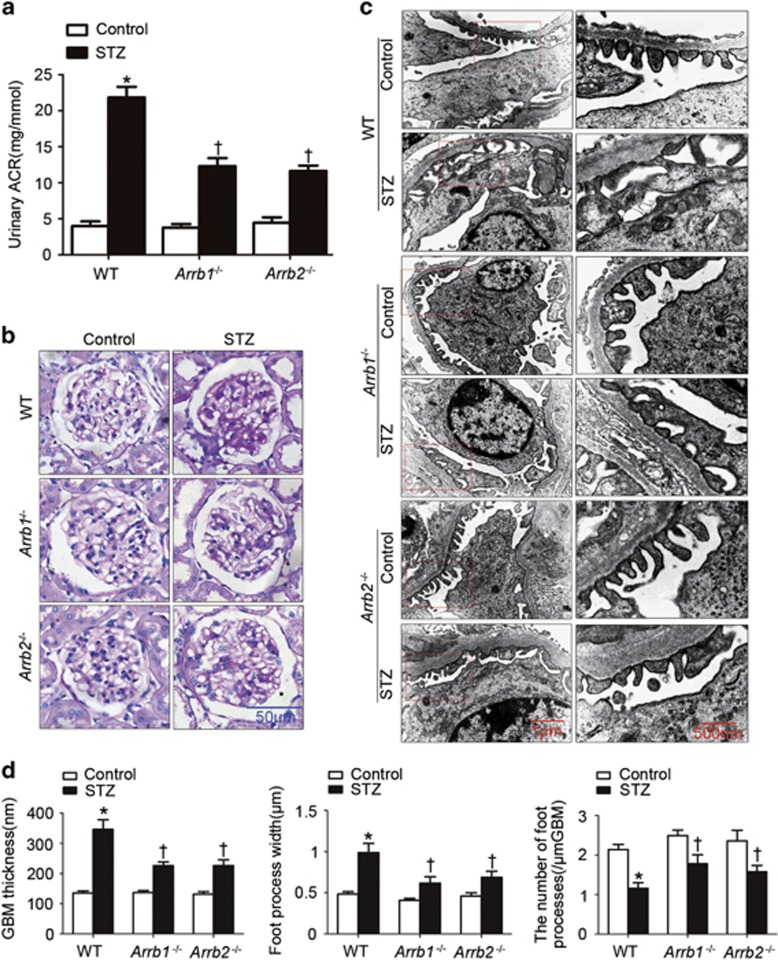
Either *Arrb1* or *Arrb2* deficiency ameliorated renal injury in diabetic mice. (**a**) Urinary ACR in different groups of mice. (**b**) Representative photomicrographs showing typical glomerular structure changes in different groups of mice. (**c**) Representative TEM images showing morphological changes in the podocyte foot process in different groups of mice. (**d**) Indices for glomerular filtration barrier integrity, including GBM thickness, foot process width and the number of foot processes*/μ*m GBM (mean±S.E.M.; *n*=8; *P<0.05 *versus* control, †*P*<0.05 *versus* WT diabetic mice

As shown in Figure 2a, urinary albumin-to-creatinine ratio (ACR) was significantly reduced in uninephrectomized (Unx) *Arrb1* or *Arrb2*-deficient (*Arrb1*^*−/−*^ *or Arrb2*^*−/−*^) diabetic mice. In wild-type (WT) diabetic mice, morphological examinations showed that the glomerular mesangium was expanded with hypercellularity and capillary collapse in the glomerulus (Figure 2b). Transmission electron microscopy (TEM) analyses further revealed podocyte injuries in diabetic mice (Figures 2c and d). The glomerular basement membrane (GBM) under the foot process effacement was thickened and the three-layered structure was lost. The number of foot processes along the GBM that was significantly decreased paralleled the increase in podocyte foot process width in diabetic mice. All of which could be ameliorated by *Arrb1 or Arrb2* deficiency.

### *β*-Arrestin-1 and *β*-arrestin-2 were upregulated in podocytes under hyperglycemia condition both *in vivo* and *in vitro*

**Figure 3:**
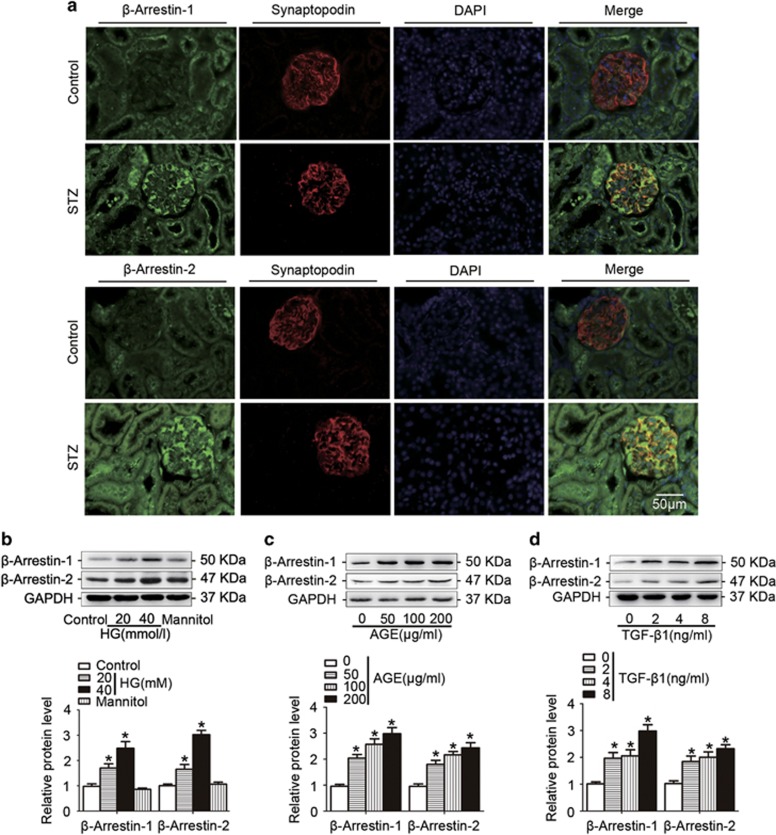
*β*-Arrestin-1 and *β*-arrestin-2 were upregulated in podocytes under hyperglycemia both *in vivo* and *in vitro*. (**a**) Representative confocal microscopic images showing the upregulation of podocyte *β*-arrestin-1/2 in the kidney from STZ-induced diabetic mice; synaptopodin was used as a podocyte marker. (**b**) Representative western blotting gel documents and summarized data showing the protein levels of *β*-arrestin-1/2 in podocytes treated with HG (final concentration 20 or 40 mmol/l in medium) for 24 h. (**c**) Representative western blotting gel documents and summarized data showing the protein levels of *β*-arrestin-1/2 in podocytes with AGE (50–200 *μ*g/ml) for 24 h. (**d**) Representative western blotting gel documents and summarized data showing the protein levels of *β*-arrestin-1/2 in podocytes treated with TGF-*β*1 (2–8 ng/ml) for 24 h. (means±S.E.M.; *n*=6; *P<0.05 *versus* control)

First, immunofluorescent results further confirmed that both *β*-arrestin-1 and *β*-arrestin-2 expression levels were increased in podocytes from diabetic mice (Figure 3a). We then detected the expression patterns in podocytes treated with high glucose (HG, Figure 3b), advanced glycation end-products (AGEs, Figure 3c) or transforming growth factor-*β*1 (TGF-*β*1, Figure 3d) (the common detrimental factors in DN) *in vitro*. Our results revealed that all these stimuli significantly increased the expression levels of podocyte *β*-arrestin-1 and *β*-arrestin-2 in a concentration-dependent manner.

### *β*-Arrestin-1 and *β*-arrestin-2 reduced basal autophagy in podocytes with HG treatment

**Figure 4:**
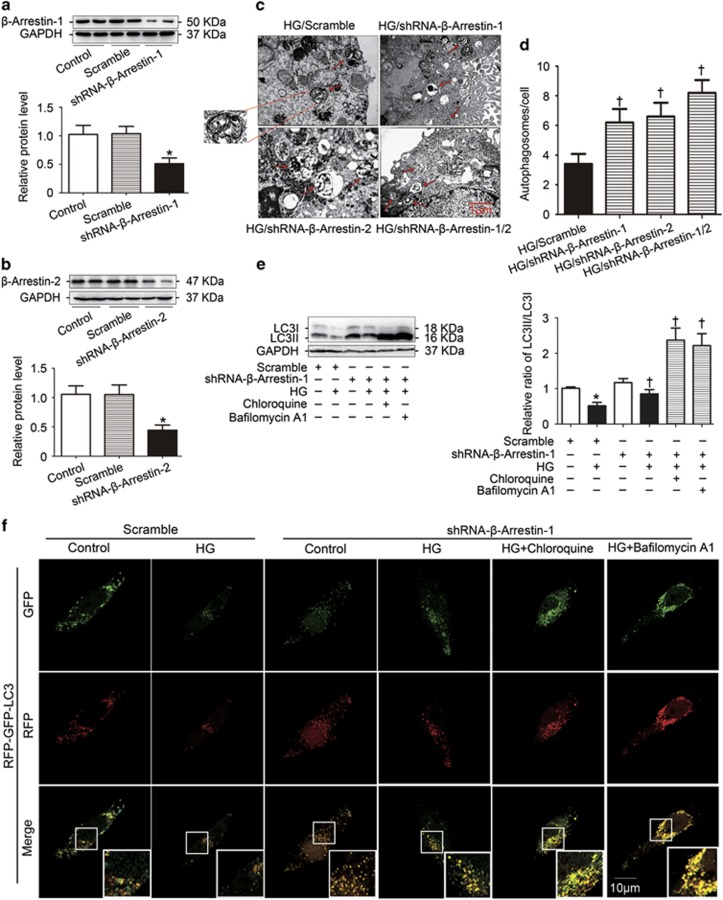
*β*-Arrestin-1 and *β*-arrestin-2 reduced basal autophagy in podocytes with HG treatment. (**a**) Representative western blotting gel documents and summarized data showing the efficiency of *Arrb1* knockdown by shRNA-*β*-arrestin-1 transfection. (**b**) Representative western blotting gel documents and summarized data showing the efficiency of *Arrb2* knockdown by shRNA-*β*-arrestin-2 transfection. (**c**) Representative electronic micrographs showing autophagosomes in HG-treated podocytes with or without gene silencing of *Arrb1* or (and) *Arrb2*. The arrows indicate autophagosomes. (**d**) The number of autophagic vesicles (AV) was determined with 10 cells in each sample, respectively. (**e**) Representative western blotting gel documents and summarized data show the levels of LC3-II/LC3-I in the presence of lysosomal inhibitors such as chloroquine (30*μ*mol/l) and bafilomycin A1 (50 nmol/l) after *Arrb1* knockdown. (**f**) Representative images of LC3 staining by measurement of fluorescent intensity in podocytes in different groups of podocytes infected with RFP–GFP–LC3 adenovirus for 24 h (means±S.E.M.; *n*=6; *P<0.05 *versus* control, †*P*<0.05 *versus* scramble of HG treatment)

To investigate the role of *β*-arrestins on the regulation of autophage in podocytes, gene silencing of *Arrb1* (Figure 4a) or *Arrb2* (Figure 4b) were used in this study. TEM showed that the number of typical autophagosomes with double membranes was significantly increased by gene silencing of *Arrb1* or (and) *Arrb2* in podocytes with HG treatment (Figures 4c and d). Moreover, autophagic flux is often inferred on the basis of LC3-II turnover, measured by western blotting in the presence of lysosomal inhibitors such as chloroquine and bafilomycin A1 that elevate/neutralize the lysosomal/vacuolar pH. We found that gene silencing of *Arrb1* increased the LC3-II/LC3-I conversion and the amount of LC3-II was much higher in the presence of chloroquine and bafilomycin A1, indicating that autophagic flux was occurring (Figure 4e). Finally, we utilized the tandem RFP-GFP-LC3 adenovirus construct to monitor autophagic flux as described.^7, 13^ This assay is based on the pH difference between the acidic autolysosome and the neutral autophagosome. In addition, green fluorescent protein (GFP) signal is sensitive to the acidic and/or proteolytic conditions of the lysosome lumen, whereas red fluorescent protein (RFP) is relatively stable. Therefore, colocalization of both GFP and RFP fluorescence indicates a compartment that has not fused with a lysosome, such as the phagophore or an autophagosome. When an autophagosome fuses with a lysosome to form autolysosomes, the GFP moiety degrades. In Figure 4f, we observed the successful introduction of the RFP-GFP-LC3 adenovirus construct showing both fluorescent proteins. In addition to accumulation of LC3, more red puncta were present in podocytes transfected with shRNA-*β*-arrestin-1 under HG conditions. In the presence of chloroquine and bafilomycin A1, GFP-LC3-positive structures and yellow signals that results from merging the red and green channels were further increased. Similar results were also found from podocytes transfected with shRNA-*β*-arrestin-2 (data not shown).

### Autophagy inhibition by *β*-arrestins reduced podocyte viability

**Figure 5:**
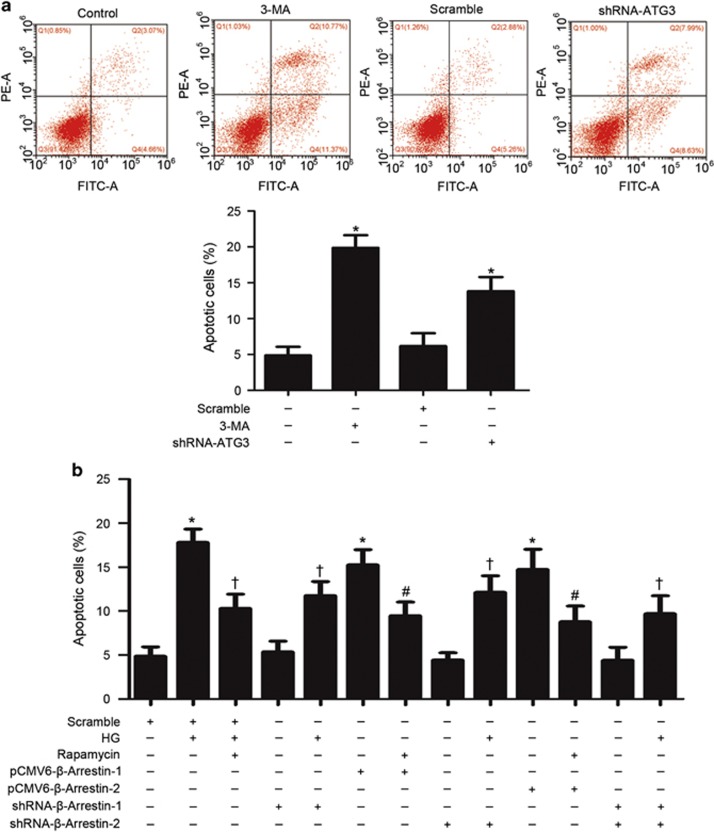
Autophagy inhibition by *β*-arrestins reduced podocyte viability by flow cytometric analysis. (**a**) Autophagy inhibition by gene silencing of ATG-3- or 3-methyladenine (3-MA)-induced apoptosis. (**b**) HG-induced apoptosis was alleviated by gene silencing of *β*-arrestins as well as by restoring defective autophagy with low dose of rapamycin. Overexpression of *β*-arrestins by pCMV6-*β*-arrestin-1 or pCMV6-*β*-arrestin-2 transfection in podocytes induced apoptosis, which could be attenuated by rapamycin. (means±S.E.M.; *n*=6; *P<0.05 *versus* control, †*P*<0.05 *versus* scramble of HG treatment, #*P*<0.05 *versus* overexpression of *β*-arrestins treatment)

To examine the role of autophagy in podocytes, autophagy inhibition by gene silencing of ATG-3 or 3-methyladenine (3-MA), an autophagy inhibitor, was used in this study. We found that autophagy inhibition induced apoptosis by flow cytometric analysis, indicating that autophagy has beneficial effects on the viability of podocytes (Figure 5a). Furthermore, we examined whether *β*-arrestins regulate podocyte viability via autophagy, and an autophagy enhancer rapamycin was used as a control to restore autophagy. Our study showed that HG-induced apoptosis was alleviated by gene silencing of *β*-arrestins as well as by restoring defective autophagy with low dose of rapamycin. Consistently, overexpression of *β*-arrestins induced apoptosis, which could be attenuated by rapamycin. Collectively, our results suggested that basal autophagy is essential in maintaining podocyte viability and that the inhibition of autophagy by *β*-arrestins may have a severe negative impact on podocyte function.

### *β*-Arrestin-1 and *β*-arrestin-2 were interacted with beclin-1 and VPS34 individually, but had no effects on the formation of PI3K core complex in podocytes under HG condition

**Figure 6:**
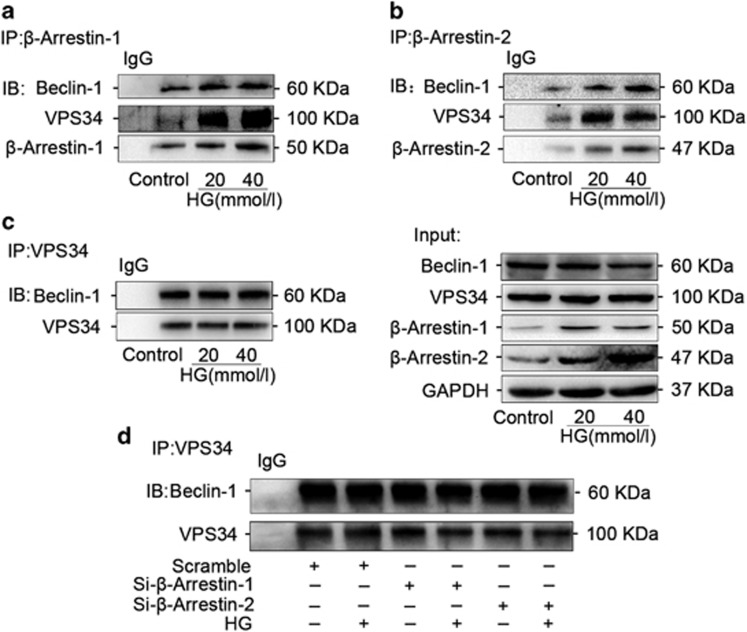
*β*-Arrestin-1 and *β*-arrestin-2 had not participated in the formation of PI3K core complex. (**a**) Immunoprecipitation assays showing that *β*-arrestin-1 interacted with VPS34 and beclin-1 and the interaction between VPS34 and *β*-arrestin-1 was significantly enhanced in podocytes with HG treatment. (**b**) An increased tendency was also observed for the interaction between VPS34 and *β*-arrestin-2. (**c**) There was no interaction change between beclin-1 and VPS34 in podocytes with HG treatment. (**d**) Gene silencing of *Arrb1* or *Arrb2* had no effects on the formation of PI3K core complex in podocytes

To explore the mechanisms by which *β*-arrestins mediated autophagy, we first investigated whether the class III PI3K core complex, which is required for autophagosome formation, can be regulated by *β*-arrestins. In this complex, VPS34 is the class III PI3K that phosphorylates phosphatidylinositol to generate phosphatidylinositol 3-phosphate, which is essential for both intracellular trafficking and autophagosome formation, and beclin 1 is part of a class III PI3K complex and participates in autophagosome formation by regulation of VPS34 activity.^14^ Immunoprecipitation assay showed that *β*-arrestin-1 interacted with VPS34 and beclin-1 and the interaction between VPS34 and *β*-arrestin-1 was significantly enhanced in podocytes with HG treatment (Figure 6a). Similarly, an increased tendency was also observed for the interaction between VPS34 and *β*-arrestin-2 (Figure 6b). However, there was no interaction change between belcin-1 and VPS34 in podocytes with HG treatment (Figure 6c) as well as gene silencing of *Arrb1* or *Arrb2* (Figure 6d). These results indicated that neither *β*-arrestin-1 nor *β*-arrestin-2 had effects on the formation of PI3K core complex under HG condition in podocytes.

### *β*-Arrestin-1 and *β*-arrestin-2 negatively regulated ATG12–ATG5 conjugation system in podocytes with HG treatment

**Figure 7:**
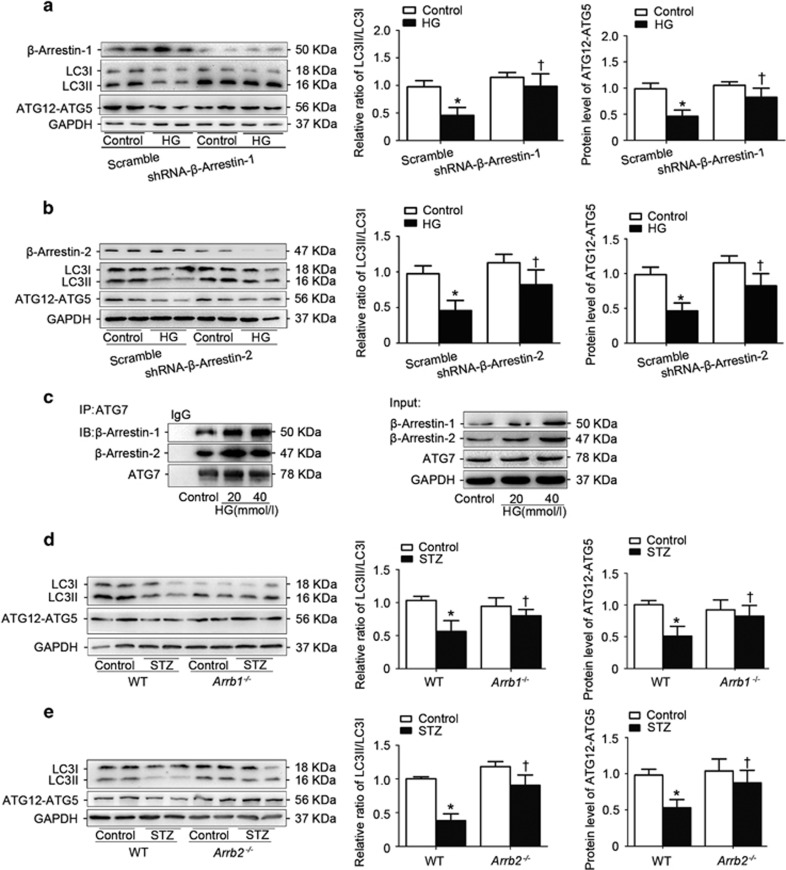
*β*-Arrestin-1 and *β*-arrestin-2 negatively regulated ATG12–ATG5 conjugation system in podocytes with HG treatment. (**a**) Representative western blotting gel documents and summarized data showing the effect of *Arrb1* knockdown on the levels of ATG12–ATG5 and LC3-II/LC3-I. (**b**) Representative western blotting gel documents and summarized data showing the effect of *Arrb2* knockdown on the levels of ATG12–ATG5 and LC3-II/LC3-I. (**c**) Immunoprecipitation assay showing that *β*-arrestin-1/2 interacted with ATG7 and the interactions between ATG7 and *β*-arrestin-1/2 were significantly enhanced in podocytes with HG treatment. (means±S.E.M.; *n*=6; *P<0.05 *versus* control, †*P*<0.05 *versus* scramble of HG treatment). (**d**) Representative western blotting gel documents and summarized data showing the levels of ATG12–ATG5 and LC3-II/LC3-I in the kidney from *Arrb1*^*−/−*^ diabetic mice. (**e**) Representative western blotting gel documents and summarized data showing the levels of ATG12–ATG5 and LC3-II/LC3-I in the kidney from *Arrb2*^*−/−*^ diabetic mice. Values are means±S.E.M.; *P<0.05 *versus* control, †*P*<0.05 *versus* WT STZ mice (*n*=8)

The LC3-II/LC3-I ratio usually correlates with the bulk autophagic flux and autophagosome formation, and the levels of the ATG12–ATG5 conjugate is used as a measure of effectiveness in early stages of autophagy leading up to autophagosome formation.^15^ Therefore, we further detected the effect of *β*-arrestins on ATG12–ATG5 conjugation and LC3 processing. It was found that HG dramatically reduced ATG12–ATG5 conjugation and LC3-II/LC3-I ratio, which can be restored by gene silencing of *Arrb1* (Figure 7a) or *Arrb2* (Figure 7b). Consistently, overexpression of *Arrb1* (Supplementary Figure S2a) or *Arrb2* (Supplementary Figure S2b) significantly reduced ATG12–ATG5 levels and LC3-II/LC3-I ratio. These results further confirmed the role of *β*-arrestins in autophagosome formation. Notably, ATG7, an E1-like enzyme that is essential for ATG12–ATG5 conjugation and LC3-II/LC3-I conversion, interacted with *β*-arrestin-1 and *β*-arrestin-2 (Figure 7c), which could be strengthened by HG treatment. Consistently, we found that the LC3-II/LC3-I ratio and ATG12-ATG5 levels were recovered in the kidney of *Arrb1*^*−/−*^ (Figure 7d) or *Arrb2*^*−/−*^ (Figure 7e) diabetic mice compared with those of WT diabetic mice *in vivo*.

## Discussion

In this study, we identified for the first time that *β*-arrestin-1 and *β*-arrestin-2 are upregulated in DN and that the defective autophagy and the impaired filtration barrier function in diabetic mice are alleviated by *Arrb1* or *Arrb2* deficiency. Although we have not examined the protective effects in the *Arrb1*^*−/−*^*/Arrb2*^*−/−*^ double knockout mice because of embryonic lethality of these mice, these results clearly indicate the importance of *β*-arrestin-1 and *β*-arrestin-2 in mediating autophagy and renal function. Considering that podocyte function as GBM turnover, maintenance of the glomerular filtration barrier and regulation of glomerular filtration,^16, 17^ and the high level of basal autophagy in podocytes is vital to maintain their architectural integrity,^3^ we therefore focus on the role of *β*-arrestins in diabetic-induced podocyte injury. Consistently, both *β*-arrestin-1 and *β*-arrestin-2 are associated with hyperglycaemia-reduced autophagy in podocytes *in vitro*.

**Figure 8:**
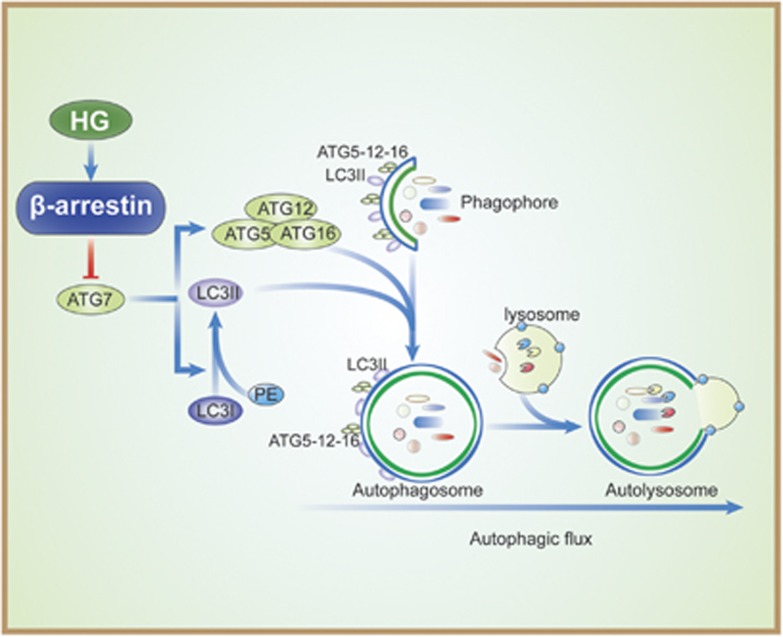
Proposed mechanisms by which *β*-arrestins negatively mediate podocyte autophagic process in DN. The enhanced interaction between *β*-arrestin-1/2 and ATG7 may block ATG7 to freely access and activate glycine residue of ATG12, thereby reducing ATG12–ATG5 conjugation, leading to the reduction of autophagy

The regulation of autophagy is extremely complicated and multiple signaling pathways are involved in this process. ATGs are key molecules in the core of the regulatory machinery of autophagy.^18^ Among them, the class III PI3K complex (beclin-1-ATG14-VPS15-VPS34) has an essential role in the initiation of the autophagosome formation. Mechanically, class III PI3K, also named VPS34, is associated with and regulated by beclin-1, and the interaction of beclin-1 with VPS34 promotes the catalytic activity of VPS34, which is crucial for canonical autophagosome formation.^19^ Several cofactors have been identified to interact with beclin-1, such as ATG14L, Bif-1, Rubicon and Bcl-2, to regulate VPS34 activity either positively or negatively.^20^ A recent study has shown that *β*-arrestin-1 interacts with beclin-1 and VPS34 under hypoxia condition but not under normal conditions in neurons, which contributes to promote autophagosome formation by enhanced beclin-1 and VPS34 interaction,^21^ thereby inducing autophagy. However, the present study displays different outcomes under hyperglycemia condition. Although we found that *β*-arrestin-1 as well as *β*-arrestin-2 interacts with beclin-1 and VPS34 even in the normal condition, and *β*-arrestin-1 and *β*-arrestin-2 are further recruited to the PI3K core complex under HG condition, there is no effect on the interaction between beclin-1 and VPS34. The mixed observation may result from the possible different contribution of *β*-arrestins to autophagy in different diseases or different cell types. So how *β*-arrestins negatively regulate autophagy in podocytes is an intriguing question of fundamental importance. In this study, we provide potential mechanisms to answer this question. The elongation and expansion steps in autophagosome formation involve two ubiquitin-like proteins, ATG12 and ATG8/LC3. The conjugation of ATG12 to ATG5 is catalyzed by ATG7 and ATG10 (E1- and E2-like enzymes, respectively) to form covalently linked ATG12–ATG5.^22^ Following formation of the ATG12–ATG5 conjugate, ATG16L non-covalently associates with this conjugate to produce the ATG12–ATG5–ATG16 multimeric complex,^23^ which is essential for LC3-PE conjugation and proper elongation of the isolation membrane. In addition, ATG7 also interacts with E2-like enzyme, ATG3, to mediate conjugation of ATG8/LC3, which is a key step during the expansion of phagophores in autophagy.^24^ Therefore, ATG7 takes a center stage on the regulation of these two conjugation systems. Interestingly, we find that HG-reduced ATG12–ATG5 levels, as well as LC3-II/LC3-I ratio, can be recovered by gene silencing of *Arrb1* or *Arrb2*. Furthermore, we observe that both *β*-arrestin-1 and *β*-arrestin-2 interacted with ATG7, individually, which can be enhanced by HG treatment. Therefore, we propose that the enhanced interaction between *β*-arrestin-1/2 and ATG7 may block ATG7 to freely access and activate glycine residue of ATG12, thereby reducing ATG12–ATG5 conjugation and suppressing autophagy (Figure 8). Further studies are needed to clarify the precise mechanisms bridging autophagy and *β*-arrestin-1/2 through mediating the enzymatic activity of ATG7.

It should be noted that, although we focus on podocyte function regulated by *β*-arrestins in DN, the upregulation was also observed in both proximal tubules and distal tubules in the kidney from diabetic mice and patients. Recent studies have found that tubular epithelial cells such as HK-2 and LLC-PK1 (porcine) subjected to HG exhibited inhibition of autophagy and altered expression of mitophagic proteins.^25^ Therefore, it is necessary to further clarify whether autophagy in tubular epithelial cells shares the same mechanisms as podocytes regulated by *β*-arrestins in DN.

In addition, although in this study we demonstrated that *β*-arrestin-1 and *β*-arrestin-2 suppress podocyte autophagy by negative regulation of ATG12–ATG5 conjugation, thereby resulting in podocyte injury, we cannot exclude that other mechanisms are also involved in the regulation of podocyte function. Wnt/*β*-catenin signaling cascade exhibits a pivotal function in the progression of DN and other proteinuric kidney diseases.^26, 27, 28, 29^ Our preliminary studies have also found that phosphoprotein disheveled 2 (DVL-2), an integral part of Wnt signaling,^30^ is associated with and upregulated by *β*-arrestins in podocytes with HG treatment. This association between *β*-arrestins and DVL-2 inhibits *β*-catenin degradation, thereby activating Wnt/*β*-catenin signaling (unpublished data). Considering recent studies indicating that Wnt/*β*-catenin signaling has a central role in mediating podocyte dysfunction and proteinuria by controlling the rennin–angiotensin system and Snail signaling,^31^ it is possible that *β*-arrestin–DVL-2-Wnt/*β*-catenin axis besides autophagy inhibition may promote podocyte injuries in DN. Moreover, GPCRs have attracted considerable attention in DN. Numerous GPCRs such as receptors for angiotensin II, endothelin, thromboxane and E-series prostaglandins have been identified in podocytes and implicated in the progression of glomerular diseases. GPCR-activated signaling pathways are associated with the activation of phospholipase C through Gαq. Wang *et al.*^32^ demonstrated that the Gαq-coupled signaling pathways in glomerular podocytes promoted renal injury. In addition, G*α* 12 couples to mumerous GPCRs and regulates multiple epithelial responses such as apoptosis, permeability and the actin cytoskeleton. Recent studies found that G*α* 12 activation in podocytes led to proteinuria and glomerulosclerosis via regulating collagen IV.^33^ Considering that *β*-arrestins are originally identified as negative adaptors of GPCRs and a very recent study reveals that *β*-arrestin-1 drives endothelin-1-mediated podocyte activation and sustains renal injury in adriamycin-induced nephropathy,^34^ it is very possible that *β*-arrestins may also regulate GPCR signaling in podocytes by regulation of their desensitization and internalization, thereby leading to podocyte injury. However, how *β*-arrestins regulate GPCR in podocyte pathology under disease conditions remain to be investigated.

In conclusion, this study for the first time explores the role of *β*-arrestins in DN and provides direct evidence that *β*-arrestin-1 and *β*-arrestin-2 share common mechanisms to mediate podocyte autophagy by negative regulation of ATG12–ATG5 conjugation, indicating that *β*-arrestins are critical components of multiple signal transduction pathways that link renal injury to reduced autophagy in DN. Modulation of these pathways may be an innovative therapeutic strategy for treating patients with DN.

## Materials and Methods

### Animal studies

The *Arrb1*- and *Arrb2*-deficient mice (*Arrb1*^*−/−*^ and *Arrb2*^*−/−*^) were originally produced by Dr. Lefkowitz's laboratory at Duke University (Durham, NC, USA) as described,^35, 36^ which have been crossed back to C57BL/6 mice for >16 generation. After transferring to Shandong University, these mice are crossed with C57BL/6 genetic mice for >10 generations. Mouse models of diabetes were developed by induction of streptozotocin (STZ) into 10-week-old male *Arrb1*^*−/−*^, *Arrb2*^*−/−*^ or WT C57BL/6 mice. Because mice with a C57BL/6 background do not develop lesions of DN readily after the induction of diabetes by STZ, Unx was performed to hasten the development of DN following previous studies.^37^ Briefly, after a 1-week recovery period from Unx, diabetes was induced by intraperitoneal injection of STZ (100 mg/kg body weight for 3 consecutive days). An equivalent amount of sodium citrate buffer alone was used as a vehicle control. After mice were injected with an intraperitoneal injection of STZ, blood glucose levels were monitored 48 h later and periodically thereafter (LifeScan One Touch glucometer, Johnson & Johnson, Milpitas, CA, USA) by mice-tailed blood sampling. Mice with blood glucose levels >15.0 mmol/l were considered as diabetic. All mice had unrestricted access to food/water and were maintained for 12 weeks in accordance with Institutional Animal Care and Use Committee procedures of Shandong University. At the end of the study, urine was collected for 24 h in a metabolic cage and urinary albumin excretion was measured using a mouse albumin ELISA Quantitation Kit (Bethyl Laboratories, Montgomery, TX, USA). Simultaneously, mice were killed under ketamine anesthesia. The fixed kidneys were paraffin embedded, and the sections were prepared and stained with periodic acid–Schiff stain. Renal tissue was homogenized for immunoblotting and mRNA analysis. The investigation conforms to the US National Institutes of Health Guide for the Care and Use of Laboratory Animals.

### *db/db* mice

Twelve-week-old male type 2 diabetic *db/db* mice and genetic control *db/+* mice were obtained from the Jackson laboratory (Bar Harbor, ME, USA) as described.^7^

### Human renal biopsy samples

Renal biopsies had been performed as part of routine clinical diagnostic investigation and collected as described in Supplementary Table S2. Among them, DM-NN were selected from patients who underwent nephrectomy for solitary renal cell carcinoma and had a concomitant diagnosis of type 2 diabetes. Histological examinations and biochemical analysis (urine ACR<30 mg/g) revealed no features of DN or other renal disease except for the solitary renal cell carcinoma. The samples of renal biopsies were obtained from the Department of Pathology, Shandong University School of Medicine, Jinan, China and the Department of Nephrology, Union Hospital, Tongji Medical College, Huazhong University of Science and Technology, Wuhan, China. Control samples were obtained from the healthy kidney poles of individuals who underwent tumor nephrectomies without diabetes or renal disease (Supplementary Table S2). The investigations were conducted in accordance with the principles of the Declaration of Helsinki and were approved by the Research Ethics Committee of Shandong University after informed consent was obtained from the patients.

### Transmission electron microscopy

Electron microscopic sample handling and detection were performed by the electron microscopic core lab of Shandong University as described.^38, 39^ TEM images were analyzed using Image J (National Institutes of Health, NIH, Bethesda, MD, USA) and analysis was based on previous studies.^40, 41^ The GBM thickness, foot process width and the number of foot processes per *μ*m of GBM were calculated using a curvimeter (SAKURAI CO., LTD, Tokyo, Japan) as described.^42^ Five glomeruli were randomly selected from each mouse and 10 electron micrographs were taken in each glomerulus.

### Cell culture and treatments

Conditionally immortalized human podocytes were originally provided by Dr. Saleem MA from University of Bristol at Bristol, UK and were cultured in RPMI 1640 medium containing 11.0 mmol/l glucose as described.^43, 44^ Different stimuli were used in this study: (1) HG and mannitol was used as the osmolarity control; to study the effect of HG, glucose at a final concentration of 20 or 40 mmol/l (additional 9.0 or 29.0 mmol/l glucose was added in the medium for podocytes) were employed in this study and mannitol (29.0 mmol/l mannitol was added in the medium for podocytes); (2) AGE (50–200 *μ*g/ml); and (3) TGF-*β*1 (2–8 ng/ml). In addition, chloroquine (30 *μ*mol/l) or bafilomycin A1 (50 nmol/l) was used to pretreat cells.

### *In situ* hybridization

Formalin-fixed, paraffin-embedded kidney tissues were sectioned (6-*μ*m thickness) and assayed for *Arrb1* and *Arrb2* RNA expression by ISH as described previously.^45, 46, 47^ *Arrb2* was detected by the *Arrb2* mRNA ISH Kit with digoxigenin (DIG)-labeled probe (Boster Biological Technology Co., Wuhan, China) according to the manufacturer's protocols. The DIG-labeled probe for *Arrb1* (5′-CATTGGTGTCAAGCTCTATGAGATTGGTGTCTACTGGAGTCTCGC-3′) was designed and synthesized by Shanghai Sunny Biotech Co., Ltd (Shanghai, China). Scramble probes (5′-CATTGGTGTCCCAGACTATGTTCAGGGTGTCTACTCATCATCTCGC-3′) were used as negative controls.

### RNA interference and overexpression of *β*-arrestins

Small interfering RNA (siRNA) to atg3 (5′-CACUUCCAGUGCCUUUCCC-3′) was synthesized by BioSune. (Jinan, China). siRNA to *Arrb1* or *Arrb2* (siRNA-*β*-arrestin-1 or siRNA-*β*-arrestin-2) was synthesized and constructed into pRNAT-U6.1/Neo to get shRNA-*β*-arrestin-2 or shRNA-*β*-arrestin-2 by Biomics Biotechnologies Co., Ltd. (Nantong, China). The DNA target sequence for shRNA-*β*-arrestin-1 is 5′-AAAGCCTTCTGTGCTGAGAAC-3′ and the DNA target sequence for shRNA-*β*-arrestin-2 is 5′-AAGGACCGGAAAGTGTTCGTG-3′. In these experiments, shRNA was transfected by Lipofectamine 2000 (Invitrogen, Gaithersburg, MD, USA) according to the manufacturer's instruction. For overexpressions of *β*-arrestin-1 and *β*-arrestin-2, podocytes were transfected with pCMV6-*β*-arrestin-1 or pCMV6-*β*-arrestin-2 plasmids by Lipofectamine 2000 as described.^48^

### RNA extraction and real-time RT-PCR

Total RNA was isolated from mouse kidney or cells using TRIzol reagent (Invitrogen) as described previously.^49^ mRNA levels for target genes were analyzed by real-time quantitative RT-PCR using a Bio-Rad iCycler system (Bio-Rad, Hercules, CA, USA). The specific primers for target genes in this study are listed in Supplementary Table S3.

### Western blotting and immunoprecipitation analyses

Total cellular lysate preparation and western blotting analysis were performed as described previously.^50^ Antibodies used in this study are summarized in Supplementary Table S4. To document the loading controls, the membrane was reprobed with a primary antibody against housekeeping protein GAPDH. For measurement of the interaction between target molecules, cellular lysates were analyzed by immunoprecipitation as described.^51^

### Immunofluorescence staining and confocal microscopy

Primary polyclonal antibodies *β*-arrestin-1 and *β*-arrestin-2 (1 : 100 dilution, ProteinTech Group, Chicago, IL, USA) were used for immunofluorescent staining, and images were obtained by a LSM780 laser scanning confocal microscope (ZEISS, Jena, Germany) system as described.^52, 37^ To monitor the various stages of autophagy, the tandem GFP-RFP-LC3 adenovirus construct obtained from Hanbio Inc. (Shanghai, China) was used in this study. This tandem GFP-RFP-LC3 construct capitalizes on the pH difference between the acidic autolysosome and the neutral autophagosome and the pH sensitivity differences exhibited by GFP and RFP to monitor progression from the autophagosome to autolysosome. Briefly, to perform image-based analysis for autophagy, podocytes were infected with the tandem GFP-RFP-LC3 adenovirus for 24 h, and then the cells were treated and imaged for GFP and RFP by using confocal fluorescence microscopy.

### Statistics

Data are expressed as means±S.E.M. The significance of the differences in mean values between and within multiple groups was examined by one-way ANOVA followed by Duncan's multiple range test. *P*<0.05 was considered statistically significant.

## Abbreviations

GPCR: G protein-coupled receptor
PI3K: phosphoinositide 3-kinase
VPS34: vacuolar protein sorting 34
siRNA: small interfering RNA
STZ: streptozotocin
WT: wild type
DN: diabetic nephropathy
HG: high glucose
AGE: advanced glycation end-product
TEM: transmission electron microscopy
