# Supplementary material for: β-Arrestins promote podocyte injury by inhibition of autophagy in diabetic nephropathy
Source: Cell Death Dis. 2016 Apr 7;7(4):e2183–. doi: 10.1038/cddis.2016.89 (PMC4855668; doi:10.1038/cddis.2016.89)
Supplement: Supplementary Information [file cddis201689x1.doc]

**Supplementary INFORMATION**

**β-arrestins** **promote podocyte injury by inhibition of autophagy in diabetic nephropathy**

Jiang Liu^1^, Quanxin Li^1^, Xiaojie Wang^1^, Chun Zhang^2^, Yiqi Duan^1^, Ziying Wang^1^, Yan Zhang^1^, Xiao Yu^3^, Ningjun Li^4^, Jinpeng Sun^5*^ and Fan Yi^1,6*^

^1^Department of Pharmacology, Shandong University School of Medicine, Jinan, China, 250012

^2^Department of Nephrology, Union Hospital, Tongji Medical College, Huazhong University of Science and Technology, Wuhan, China, 430022

^3^Department of Physiology, Shandong University School of Medicine, Jinan, China, 250012

^4^Department of Pharmacology and Toxicology, Medical College of Virginia, Virginia Commonwealth University, Richmond, VA 23298, USA

^5^Department of Biochemistry and Molecular Biology, Shandong University School of Medicine, Jinan, China, 250012

^6^Institute of Nephrology, Shandong University, Jinan, China, 250012

*

**Corresponding author:**

Fan Yi, Ph.D

Professor

Department of Pharmacology

Shandong University School of Medicine

44#, Wenhua Xi Road,

Jinan, Shandong, 250012, P.R. China

Phone: 86-0531-88382616

Fax: 86-0531-88382616

E-mail: [fanyi@sdu.edu.cn](mailto:fanyi@sdu.edu.cn)

or

Jinpeng Sun, Ph.D

Professor

Department of Biochemistry and Molecular Biology,

Shandong University School of Medicine

44#, Wenhua Xi Road,

Jinan, Shandong, 250012, P.R. China

Phone: 86-0531-88382616

Fax: 86-0531-88382616

E-mail: [sunjinpeng@sdu.edu.cn](mailto:sunjinpeng@sdu.edu.cn)

**Supplementary Table 1. Physical and biochemical parameters of experimental animals.**

| **Variable** | | **Wild-type mice** | | ***Arrb1^-/-^* mice** | | ***Arrb2^-/-^* mice** | |
| --- | --- | --- | --- | --- | --- | --- | --- |
|  |  | **Normal-Diet** | **STZ** | **Normal-diet** | **STZ** | **Normal-diet** | **STZ** |
| **Body weight (g)** | | 29.1 ± 2.33 | 21.4 ± 1.72 | 27.4 ± 1.36 | 20.6 ± 1.571 | 28.9 ± 0.92 | 21.8 ± 1.89 |
| **Heart rate (beat/min)** | | 453.6 ± 9.37 | 447.1 ±7.20 | 473.1 ± 11.31 | 466.8 ± 9.19 | 459.2 ± 10.16 | 471.3 ± 8.32 |
| **Blood pressure (mmHg)** | **Systolic** | 103.0 ± 9.7 | 105.7 ± 10.3 | 104.9 ± 9.1 | 107.3 ± 12.6 | 104.3 ± 8.0 | 105.1 ± 10.7 |
|  | **Diastolic** | 70.2 ± 8.9 | 71.6 ± 10.4 | 73.1 ± 14.1 | 69.8 ± 7.9 | 71.1 ± 9.7 | 70.3 ± 10.8 |
| **Glucose (mM)** | | 5.9 ± 0.91 | 20.3 ± 2.33* | 6.2 ± 1.04 | 19.1 ± 1.94* | 6.5 ± 1.49 | 19.9 ± 1.98* |
| **Relative kidney weight (mg/g body weight)** | | 10.37 ± 0.22 | 19.75 ± 1.44* | 11.28 ± 2.10 | 16.4 ± 1.74*^†^ | 11.10 ± 0.60 | 15.2 ± 1.22*^†^ |

Male mice were uninephrectomized. After a 1-week recovery period from uninephrectomy, diabetes was induced by intraperitoneal injection of streptozotocin (100 mg/kg body weight for three consecutive days). All mice had unrestricted access to food/water and were maintained for 12 weeks. Values are means ± S.E.M. for 8 mice in each group. **P*<0.05 vs. normal-diet mice; †*P*<0.05 vs. STZ-induced diabetic wild-type mice.

**Supplementary Table 2. Clinical data and relative mRNA levels of *ARRB1 and ARRB2* in the kidney from diabetic subjects with or without DN.**

| **Patient** | **Age at Biopsy(yr)** | **Sex** | **Scr (μmol/L)** | **Serum Urea (mmol/L)** | **UPE/24h (g)** | **eGFR (ml/min/1.73m2)** | **HbA1c (%)** | **DPN** | **DR** | **IHD** | **CVA** | **Relative *ARRB1* mRNA level** | **Relative *ARRB2* mRNA level** |
| --- | --- | --- | --- | --- | --- | --- | --- | --- | --- | --- | --- | --- | --- |
| **Normal controls** |  |  |  |  |  |  |  |  |  |  |  |  |  |
| 1 | 57 | F | 65 | 3.98 | <0.15 | **105** | NA | N | N | N | N | **1.00** | **1.00** |
| 2 | 49 | M | 76 | 4.76 | <0.15 | **100** | NA | N | N | N | N | **1.32** | **0.79** |
| 3 | 68 | F | 61 | 4.62 | <0.15 | **90** | NA | N | N | N | N | **0.92** | **1.21** |
| 4 | 63 | M | 59 | 5.22 | <0.15 | **128** | NA | N | N | N | N | **0.59** | **0.77** |
| 5 | 72 | M | 92 | 7.11 | <0.15 | **75** | NA | N | N | N | N | **2.12** | **1.89** |
| 6 | 47 | M | 57 | 3.79 | <0.15 | **141** | NA | N | N | N | N | **1.52** | **1.31** |
| 7 | 61 | M | 62 | 4.77 | <0.15 | **76** | NA | N | N | N | N | **3.01** | **1.65** |
| 8 | 70 | M | 74 | 6.11 | <0.15 | **122** | NA | N | N | N | N | **0.78** | **0.47** |
| 9 | 56 | M | 69 | 4.09 | <0.15 | **109** | NA | N | N | N | N | **0.62** | **1.21** |
| mean±SD | 60.3±8.9 |  | 68.3±11.0 | 4.94±1.08 |  | **110.2±**  **27.8** |  |  |  |  |  | **1.32±0.80** | **1.14±0.45** |
| **DM-NN group** |  |  |  |  |  |  |  |  |  |  |  |  |  |
| 1 | 78 | M | 53 | 6.17 | <0.15 | **139** | 5.3 | N | N | N | Y | **0.71** | **0.86** |
| 2 | 59 | F | 72 | 4.08 | <0.15 | **76** | 7.6 | N | Y | Y | N | **1.78** | **2.11** |
| 3 | 47 | M | 89 | 4.67 | <0.15 | **84** | 6.7 | Y | N | N | N | **1.37** | **1.45** |
| 4 | 63 | M | 51 | 5.29 | <0.15 | **151** | 8.2 | N | N | N | Y | **1.11** | **0.62** |
| 5 | 56 | F | 88 | 5.01 | <0.15 | **61** | 12.7 | N | Y | Y | N | **2.98** | **1.09** |
| 6 | 72 | M | 56 | 6.02 | <0.15 | **132** | 4.8 | N | N | N | Y | **1.37** | **1.56** |
| 7 | 49 | M | 71 | 4.90 | <0.15 | **109** | 9.9 | N | Y | N | Y | **1.05** | **0.57** |
| 8 | 56 | F | 68 | 5.87 | <0.15 | **83** | 10.1 | N | Y | N | N | **1.51** | **2.78** |
| mean±SD | 60.1±10.7 |  | 76.7±14.8 | 5.25±0.73 |  | **107.7±**  **35.4** | 8.2±2.7 |  |  |  |  | **1.49±0.68** | **1.38±0.76** |
| **DN group** |  |  |  |  |  |  |  |  |  |  |  |  |  |
| 1 | 43 | M | 162 | 9.70 | 3.25 | **43** | 9.2 | Y | N | Y | N | **3.92** | **2.62** |
| 2 | 62 | M | 218 | 16.22 | 4.11 | **28** | 7.1 | N | Y | Y | N | **1.93** | **5.11** |
| 3 | 77 | F | 246 | 13.2 | 6.21 | **18** | 6.9 | Y | Y | Y | N | **7.22** | **3.78** |
| 4 | 82 | F | 286 | 22.0 | 5.89 | **15** | 13.5 | N | Y | N | Y | **4.12** | **4.76** |
| 5 | 53 | M | 132 | 18.81 | 3.02 | **52** | 6.8 | N | Y | N | N | **1.92** | **0.89** |
| 6 | 60 | F | 179 | 14.02 | 6.1 | **27** | 9.1 | N | N | Y | Y | **3.14** | **4.25** |
| 7 | 49 | F | 216 | 19.5 | 8.22 | **22** | 7.7 | Y | N | N | Y | **5.68** | **6.29** |
| 8 | 51 | M | 183 | 20.10 | 4.33 | **36** | 8.0 | N | Y | N | Y | **0.78** | **2.91** |
| mean±SD | 59.6±13.7 |  | 202.8±49.1 | 16.69±4.15 | 5.1±1.7 | **29.2±**  **10.6** | 8.5±2.1 |  |  |  |  | 3.59±2.12 | 3.83±1.68 |

SCr, serum creatinine; UPE, urinary protein excretion; eGFR, estimated GFR; HbA1c, hemoglobin A1c; F, female; M, male; DPN, diabetic peripheral neuropathy; DR, diabetic retinopathy; IHD, ischemic heart disease; CVA, cerebrovascular accident; N, not present; NA, not applicable/not available.

**Supplementary Table 3. Primer pairs of target genes used for real time RT-PCR in this study**

| Genes | Accession No. | Forward | Reverse |
| --- | --- | --- | --- |
| Mus β-arrestin1 | [NM_178220.3](http://www.ncbi.nlm.nih.gov/entrez/viewer.fcgi?db=nucleotide&id=79750060) | ACCTTTGAGATCCCGCCAAA | CAGGGGCATACTGAACCTTC |
| Mus β-arrestin2 | [NM_001271360.1](http://www.ncbi.nlm.nih.gov/entrez/viewer.fcgi?db=nucleotide&id=405778344) | GGAGTAGACTTTGAGATTCGAGC | CTTTCTGATGATAAGCCGCACA |
| Mus β-actin | [NM_007393.3](http://www.ncbi.nlm.nih.gov/entrez/viewer.fcgi?db=nucleotide&id=145966868) | GGCTGTATTCCCCTCCATCG | CCAGTTGGTAACAATGCCATGT |
| Homo β-arrestin1 | [NM_020251.3](http://www.ncbi.nlm.nih.gov/entrez/viewer.fcgi?db=nucleotide&id=320461704) | ATGGCCTCCTATTTATTGTTCTT | GTGTTGAGTGATTATTTTGTGACAC |
| Homo β-arrestin2 | NM_001257331 | TATGCTCAGAAACAAAGAA | GTGTTCATACCTAAATTTTCTG |
| Homo β-actin | [XM_006715764.1](http://www.ncbi.nlm.nih.gov/entrez/viewer.fcgi?db=nucleotide&id=578813593) | GAAGTGTGACGTGGACATCC | CCGATCCACACGGAGTACTT |

**Supplementary Table 4. Antibodies used in this study**

| **Primary antibodies** | **Host** | **Dilution and supplier** | **Product ID** | **Application** |
| --- | --- | --- | --- | --- |
| β-arrestin1 | Rabbit | 1:1000(1:100 for IHC, IP); Bioworld Technology, Louis Park, MN | BS2213 | WB, IF, IHC |
| β-arrestin2 | Rabbit | 1:1000 (1:100 for IHC, IP); ProteinTech Group, Chicago, IL | 10171-1-AP | WB, IF, IHC |
| ATG12-5 | Rabbit | 1:1000 (1:100 for IP); Cell Signaling, Danvers, MA | D88H11 | WB,IP |
| ATG7 | Rabbit | 1:1000 (1:100 for IP); Boster, Wuhan，China | BA3527-2 | WB,IP |
| LC3B | Rabbit | 1:1000 (1:100 for IF); Cell Signaling, Danvers, MA | 2775 | WB, IF |
| VPS34 | Rabbit | 1:1000 (1:100 for IP); ProteinTech Group, Chicago, IL | 12452-1-AP | WB, IP |
| Beclin1 | Rabbit | 1:1000; Cell Signaling, Danvers, MA | 3495 | WB |
| Synaptopodin | Goat | 1:100; Santa Cruz, Dallas, TX | sc-21536 | IF |
| GAPDH | Mouse | 1:4000; ProteinTech Group, Chicago, IL | 60004-1-Ig | WB |

**Supplementary Figure S1**

**
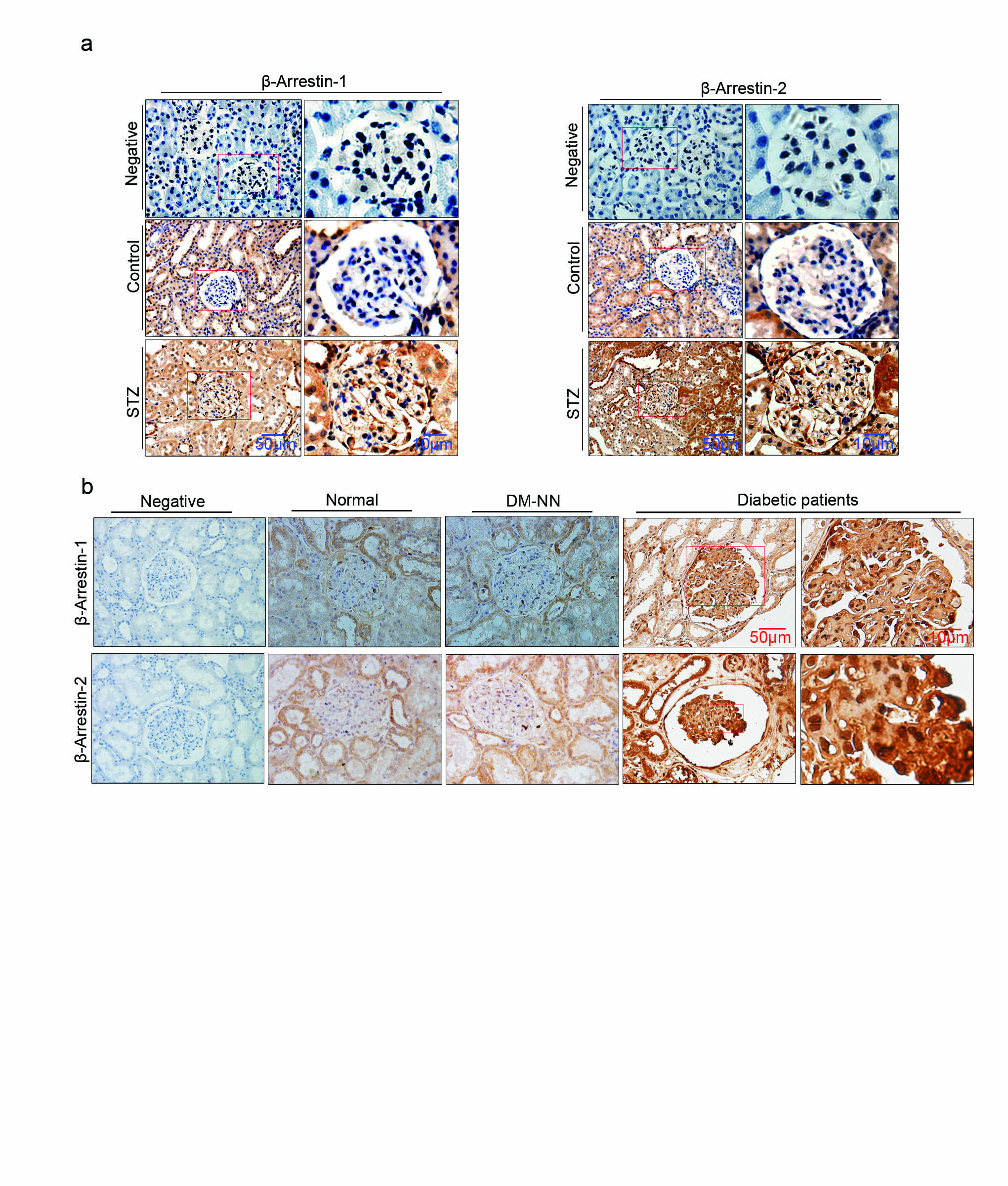
**

**Supplemental Figure S1.** **(a)** Representative photomicrographs of β-arrestin-1 and β-arrestin-2 immunohistochemical staining in the kidney from STZ-induced diabetic mice. Negative control by omission of the corresponding primary antibodies demonstrated no nonspecific staining. **P*<0.05 vs control. **(b)** Representative photomicrographs of β-arrestin-1/2 immunohistochemical staining in human renal cortical tissue from normal subjects (n=9) and patients with diabetic nephropathy (DN) (n=8) or diabetic patients without nephropathy (DM-NN) (n=8). Negative control by omission of the corresponding primary antibodies demonstrated no nonspecific staining.

**Supplementary Figure S2**

**
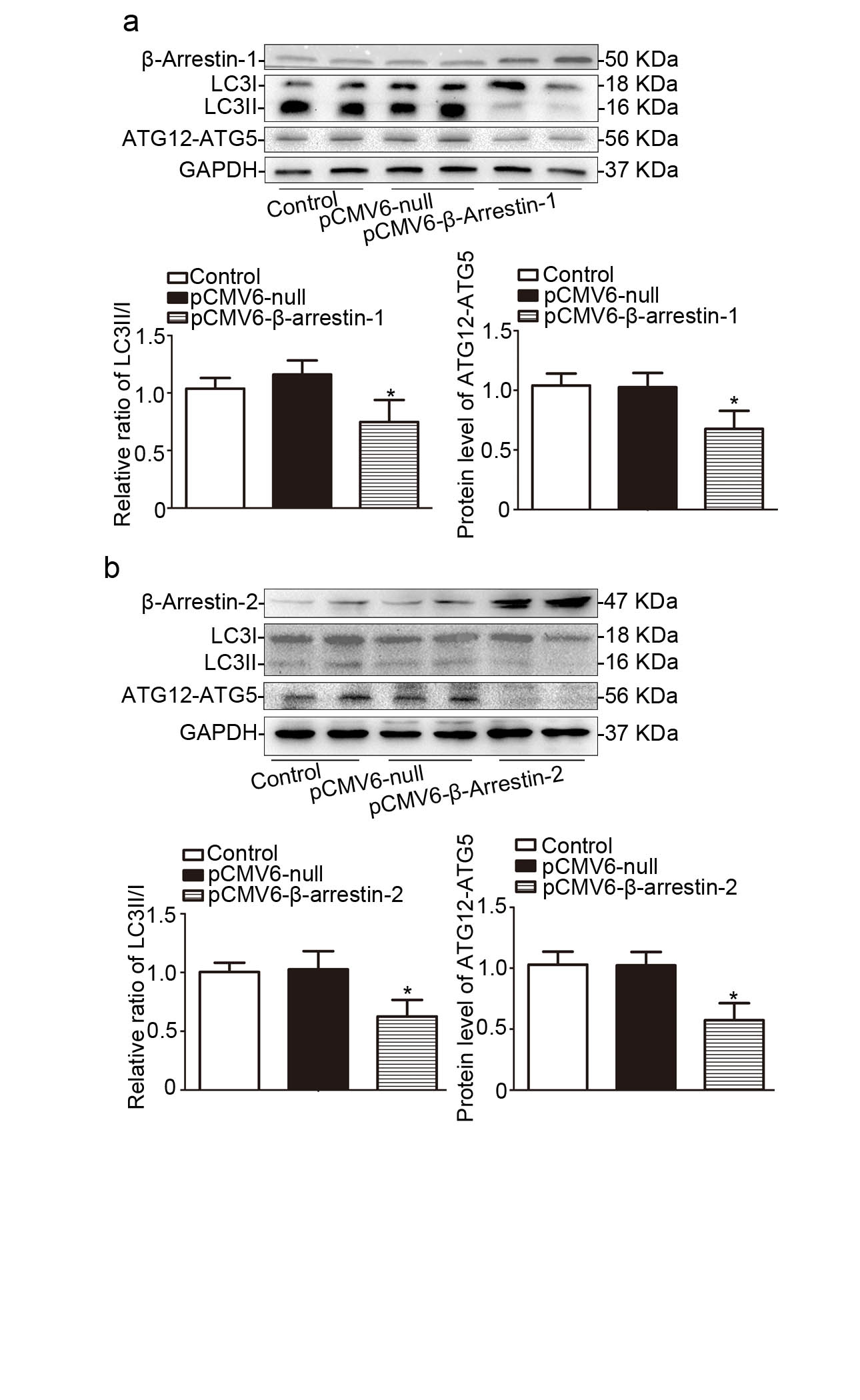
**

**Supplemental Figure S2.** **(a)** Representative Western blot gel documents and summarized data showing the effect of β-arrestin-1 overexpression on the levels of ATG12-ATG5 and LC3-II/LC3-I. **(b)** Representative Western blot gel documents and summarized data showing the effect of β-arrestin-2 overexpression on the levels of ATG12-ATG5 and LC3-II/LC3-I. Data are expressed as the means±S.E.M.; n=3, **P*<0.05 vs control
